# Spatio-temporal variations of energy carbon emissions in Xinjiang based on DMSP-OLS and NPP-VIIRS nighttime light remote sensing data

**DOI:** 10.1371/journal.pone.0312388

**Published:** 2024-10-25

**Authors:** Jie Song, Xin He, Fei Zhang, Weiwei Wang, Ngai Weng Chan, Jingchao Shi, Mou Leong Tan

**Affiliations:** 1 College of Geography and Remote Sensing Sciences, Xinjiang University, Urumqi, China; 2 College of Geography and Environmental Sciences, Zhejiang Normal University, Jinhua, China; 3 GeoInformatic Unit, Geography Section, School of Humanities, Universiti Sains Malaysia, Penang, Malaysia; 4 Departments of Earth Sciences, The University of Memphis, Memphis, TN, United States of America; Ningbo University, CHINA

## Abstract

With the rapid economic development of Xinjiang Uygur Autonomous Region (Xinjiang), energy consumption became the primary source of carbon emissions. The growth trend in energy consumption and coal-dominated energy structure are unlikely to change significantly in the short term, meaning that carbon emissions are expected to continue rising. To clarify the changes in energy-related carbon emissions in Xinjiang over the past 15 years, this paper integrates DMSP/OLS and NPP/VIIRS data to generate long-term nighttime light remote sensing data from 2005 to 2020. The data is used to analyze the distribution characteristics of carbon emissions, spatial autocorrelation, frequency of changes, and the standard deviation ellipse. The results show that: (1) From 2005 to 2020, the total carbon emissions in Xinjiang continued to grow, with noticeable urban additions although the growth rate fluctuated. In spatial distribution, non-carbon emission areas were mainly located in the northwest; low-carbon emission areas mostly small and medium-sized towns; and high-carbon emission areas were concentrated around the provincial capital and urban agglomerations. (2) There were significant regional differences in carbon emissions, with clear spatial clustering of energy consumption. The clustering stabilized, showing distinct "high-high" and "low-low" patterns. (3) Carbon emissions in central urban areas remained stable, while higher frequencies of change were seen in the peripheral areas of provincial capitals and key cities. The center of carbon emissions shifted towards southeast but later showed a trend of moving northwest. (4) Temporal and spatial variations in carbon emissions were closely linked to energy consumption intensity, population size, and economic growth. These findings provided a basis for formulating differentiated carbon emission targets and strategies, optimizing energy structures, and promoting industrial transformation to achieve low-carbon economic development in Xinjiang.

## 1 Introduction

The consumption of fossil fuels has caused severe negative environmental impacts and accelerated global warming in an increasing number of countries worldwide [1]. The Chinese government has established a range of plans for reducing carbon emissions and striving for carbon peak by 2030 and carbon neutrality by 2060 [2]. Xinjiang has a very important position in China’s energy, with reserves ranked the first in the country [3]. Interestingly, as Xinjiang’s economic growth is mainly linked to corresponding energy consumption, it has unfortunately brought serious pollution to Xinjiang’s ecological environment and led to a huge volume of resources wastes [4,5]. Consequently, it is vital to assess the variations in carbon emission levels across different regions in Xinjiang, along with the evolving patterns of these emissions over time and space, to help China meet its emissions reduction targets [6].

In recent years, remote sensing technology has the advantages in studying carbon emissions from energy consumption, and it has gradually become an important mean to study temporal and spatial changes compared to the traditional numerical analyses of Zhang et al. [7,8]. The nighttime lighting data can be utilized to estimate carbon emissions, thereby addressing the lack of energy consumption statistics in prefecture-level cities or smaller cities. By integrating the building carbon emission data from China’s eastern, central, and western regions, a regionalized model for calculating China’s building carbon emissions was established. Panel data analysis indicated a balanced correlation between the brightness values of smoothed lamps and building carbon emissions [9]. Zhang et al. [10] employed nighttime light data and statistical records from 2000 to 2019 to conduct a comparative analysis. It explored various modeling techniques, such as linear, exponential, and logarithmic models, aiming to assess carbon emissions across different spatial scales. Wang et al. [11] presents an enhanced model, ISTIRPAT (Improved Stochastic Impacts by Regression on Population, Affluence, and Technology), which is developed based on nighttime light remote-sensing data and the STIRPAT model, further incorporating the concept of the environmental Kuznets curve. In addition, Su et al. [12] analyzed the carbon emissions of typical urban agglomerations in China from 1992 to 2010 based on the fusion of nighttime lighting data, which solved the limitations of incomplete statistical data and inconsistent statistical standards. With this data fusion basis, Sun et al. [13] studied the spatial and temporal changes of carbon emissions in Chinese cities from 2000 to 2017 using nighttime satellite image data. However, although numerous studies utilize nighttime lighting data to analyze energy carbon emissions, few focused on the city level emissions and economic development in arid zones. Furthermore, the validity of using nighttime lighting data to estimate energy carbon emissions still requires further verification.

Considering this gap, it is essential to supplement the city-scale carbon emission statistical data to study the energy carbon emission characteristic of different cities in Xinjiang, a typical arid region and energy-rich province in China. In this research, long-term fused nighttime light data are obtained from DMSP-OLS and NPP-VIIRS nighttime light satellite data. The accuracy of using nighttime light data to estimate energy carbon emissions is verified by statistical data. Subsequently, the spatial and temporal changes in energy carbon emissions and their relationship with economic development in Xinjiang are further analyzed. The objectives of this study are to (1) estimate the temporal and spatial variations of carbon emission; (2) calculate the change frequency of energy-related carbon emission; (3) perform gravity center migration and standard deviation ellipse change of carbon emission; (4) analyze the correlation between energy-related carbon emission and economic growth. This study will provide a foundation for China to develop precise carbon emission reduction targets and implement low-carbon sustainable development policies.

## 2 Overview of the study area and research data

### 2.1 Study area

**Fig 1:**
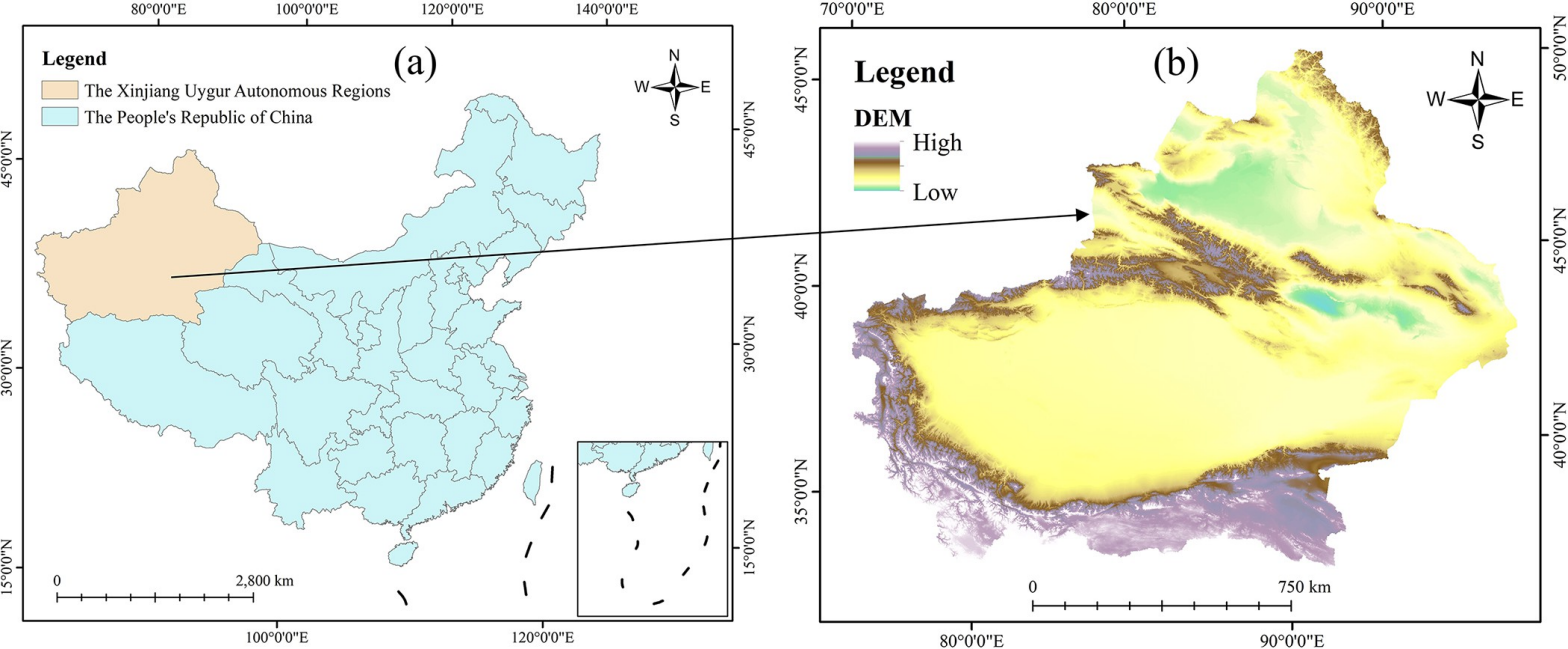
Overview map of the study area (The Review Number: GS (2024)0650): (a) The location of Xinjiang in China; (b) The elevation of the target area.

The Xinjiang Uygur Autonomous Region is located in the northwestern part of China (Fig 1), geographically between latitudes 34.37°- 49.55°N and longitudes 73.53°- 96.35°E. It is characterized as a typical temperate continental climate, with an average annual temperature of 10.40°C and an average annual precipitation of 188 mm. In recent years, Xinjiang’s economy has been developing rapidly, with a gross regional product (GDP) of 1.77 × 10^3^ billion yuan as of 2022. Xinjiang boasts abundant energy resources, including coal, oil, natural gas, as well as significant wind and solar potential [14]. Xinjiang’s fossil energy sources, particularly coal and petroleum, are high-carbon energy sources with the highest carbon content. The region’s energy consumption is heavily dependent on these high-carbon energy sources, and the lack of clean energy use contributes to the province’s hyper-carbonized character [15].

### 2.2 Data sources

This paper utilizes five primary types of data, and the details and origins of each type are presented in Table 1.

**Table 1:** Data sources in the current study.

Data name	Spatial resolution	Time duration	Data source
DMSP/OLS	1 km	2005-2013	Earth Observation Group, Payne Institute for Public Policy, Colorado School of Mines (https://eogdata.mines.edu/products/vnl)
NPP/VIIRS	0.5 km	2012-2020	
Land Scan population	1 km	2005-2020	Oak Ridge National Laboratory (ORNL), U.S. Department of Energy (https://landscan.ornl.gov)
Energy and GDP statistics	---	2005-2020	China and Xinjiang Energy Statistical Yearbook (https://www.stats.gov.cn/sj/ndsj)
Vector administrative	---	---	National Geomatics Center of China (https://www.ngcc.cn)
DEM	90 m	---	Resource and Environmental Science Data Platform (https://www.resdc.cn)

### 2.3 Data processing

Nighttime light data are fundamentally different and incompatible [16,17]. Consequently, two data still require a series of data fusion processes to be transformed into continuous, sequential and unified datasets [18]. Firstly, the nighttime light data were preprocessed before analysis. After processing, the DMSP/OLS data of 2012 and NPP/VIIRS data of 2013 were analyzed, and the optimal fitting parameters were selected after fitting these two datasets [19]. Finally, the regression analysis of the total brightness value of the municipal scale data in Xinjiang was carried out.

#### 2.3.1 Preprocessing of DMSP/OLS nighttime light data

Before data utilization, mutual correction and continuity correction are necessary. Further processing of light data can enhance its availability. The correction of saturated pixels in the night-time light data was performed using a quadratic fitted regression between the original grey values of the pixels in each region and the standard data set for each year. The correlation coefficients (R^2^) of the fits are greater than 0.75, which satisfies the requirements for establishing a regression model [20,21]. This ensures the accuracy of the model’s mutual correction. The quadratic polynomial equation used is presented as follows:

$${\mathrm{DN}}_{cal}=a\times DN^{2}+b\times DN+c$$ (1)

where *DN*_*cal*_ stands for the corrected image pixel value, *DN* represents the image to be corrected pixel value, *a* variable represents the quadratic term coefficient, *b* variable represents the primary term coefficient, and *c* variable represents the constant term coefficient.

Since each DMSP/OLS sensor has its own unique characteristics, it is inevitable that various errors will occur during the image acquisition process. Therefore, it is essential to rectify the nighttime light data from various sensors against each other. The rectification formula can be articulated as follows:

$${\mathrm{DN}}_{\left(n,i\right)}=\{\begin{array}{l} 0,DN_{\left(n,i\right)}^{a}=0\;\mathrm{or}\;DN_{\left(n,i\right)}^{b}=0\\ (DN_{\left(n,i\right)}^{a}+DN_{\left(n,i\right)}^{b})/2,other \end{array}$$ (2)

where *DN*_(*n*,*i*)_ represents the DN value of the pixel at position *i* in year *n* after correction; ${DN}_{\left(n,i\right)}^{a}$ represents the DN value of the pixel at position *i* obtained by sensor *a* in year *n*; ${DN}_{\left(n,i\right)}^{a}$ represents the DN value of the pixel at position *i* obtained by sensor *b* in year *n*; (n = 1992, 1993, 1994, …, 2013 ).

#### 2.3.2 Preprocessing of NPP/VIIRS nighttime light data

Monthly remote sensing images of night light from 2012 to 2020 were obtained by clipping the vector boundary of the resampled night light images within the research area. Based on this approach, the yearly average night light data were derived from the mean of the monthly night light data [22]. The calculation formula is stated as follows:

$$I_{m}=\sum_{i=1}^{12}\frac{I_{i}}{9}$$ (3)

where *I*_*m*_ is the synthetic annual data; and *I*_*i*_ the monthly data of the corresponding month.

NPP-VIIRS nighttime light data has maximum value and negative value, and the negative value of night light data needs to be processed, based on which, annual data is synthesized in according to monthly data.

$$\mathrm{DN}(n,i)=\{\begin{array}{l} 0,DN_{i}<0\\ DN_{i},DN_{i}>0 \end{array}$$ (4)

where *DN(n*,*i)* is the DN value of the pixel at position *i* in year *n* after correction, *DN*_*i*_ stands for the digital number value of the pixel located at position *i*.

After obtaining the annual composite data for consecutive years, it is necessary to process the extreme values of the images. This study employs a reduction method for data processing.

$$\mathrm{DN}(n,i)=\{\begin{array}{l} DN_{i},DN_{i}<DN_{\max}\\ DN_{\max},DN_{i}>DN_{\max} \end{array}$$ (5)

where *DN(n*,*i)* is the DN value of the pixel at position *i* in year *n* after processing, *DN*_*max*_ is the maximum value of the DN value of the nighttime light data.

#### 2.3.3 NPP-VIIRS fitted to DMSP-OLS

The only two years in which the two types of data coincide are 2012 and 2013, and in order to construct the long time-series nighttime light remote sensing data, it is necessary to fit the NPP/VIIRS data to the DMSP/OLS data, we presented a specific model transformation formula as follows:

$${\mathrm{DMSP}}_{\mathrm{simulate}}=F(VIIRS,a,b)\Delta M$$ (6)

where *DMSP*_*simulate*_ represents the fitted results, *F* represents the transformation function, *a* and *b* represent the estimated coefficients, Δ denotes spatial convolution, and *M* represents a normalization matrix, as shown in Eq (7).

$$G(X,a,b)=(\begin{array}{ccc} ax_{11}^{b} & \ldots & ax_{1n}^{b}\\ \vdots & \ddots & \vdots \\ ax_{m1}^{b} & \cdots & ax_{mn}^{b} \end{array})$$ (7)

$$M(x,y)=\frac{1}{2\pi \sigma^{2}}e^{\frac{\left(x^{2}+y^{2}\right)}{2\sigma^{2}}}$$ (8)

where *x*_*mn*_ represents the pixel value at the m-th row and n-th column, and the standard deviation σ is the coefficient to be estimated.

**Fig 2:**
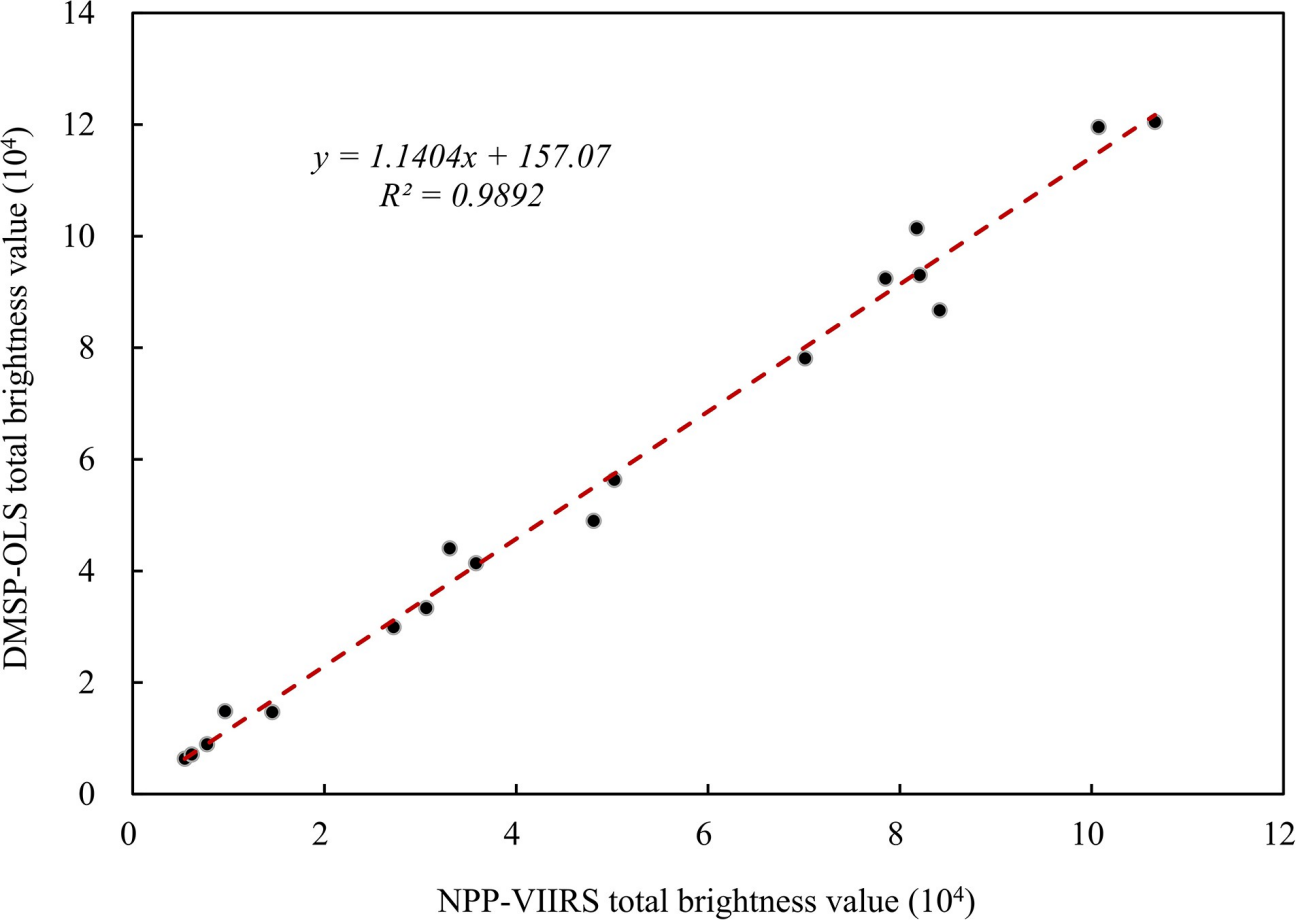
The Relationship between total digital number (TDN) of DMSP-OLS data and NPP-VIIRS data in 2012 and 2013.

Based on the nighttime light data after preprocessing, we extracted 2012 and 2013 raster pixel values from the two satellites data and then plotted scatter plots to compare the datasets within the same year. The outcomes demonstrated a strong positive association between the DMSP-OLS and NPP-VIIRS nighttime light data, as illustrated in Fig 2. The total brightness value’s R^2^ of night light data in Xinjiang from 2012 to 2013 is 0.9892, indicating a high level of comparability and continuity between the two datasets.

## 3 Research methods

### 3.1 Carbon emissions statistical analysis

This manucript selected nine types of data, to calculate Carbon emissions from energy consumption, detailed interpretation in Table 2.

**Table 2:** The mean conversion ratio for diverse categories of fuel.

Energy category	Coal	Coke	Crude	Gasoline	Kerosene	Diesel	Electricity	Natural gas	Fuel Oil
Standard coal coefficient B_i_(tce/t)	0.7143	0.9714	1.4286	1.4714	1.4714	1.4571	0.345	1.33	1.4286
Carbon emission coefficients K_i_(t/tce)	0.7476	0.1128	0.5854	0.5532	0.5931	0.3416	2.2132	0.4497	0.6176

Typically, the consumption of different fuels is standardized and then multiplied by the carbon emission factors for each fuel to calculate the actual carbon emissions for each fuel [23]. The computation is as follows:

$$CO_{2}=\frac{22}{14}\times \sum_{i=1}^{9}R_{i}E_{i}$$ (9)

where *i* represents the fossil fuel energy type; *R*_*i*_ represents the carbon emission factor of fossil fuel energy type *i*; and *E*_*i*_ represents the actual consumption of fossil fuel energy type *i* in kind.

### 3.2 Simulation of carbon emissions from energy consumption

Given the differences in per capita carbon emissions and the total brightness value-related carbon emissions across cities in Xinjiang, it is essential to perform a simulation analysis utilizing night time data, as economic development differs among these cities. The method entails estimating the carbon emission associated with the aggregate brightness value for each province annually [24], the computation is as follows:

$$\alpha_{ij}=\frac{CO_{2ij}}{POP_{\mathrm{ij}}};\;\beta_{ij}=\frac{CO_{2ij}}{DN_{ij}}$$ (10)

In the formula, *i* corresponds to distinct years, while *j* signifies different provinces.*CO*_*2ij*_ represents the carbon emissions data recorded for province *j* in year *i*.; *POP*_*ij*_ represents the total population for province *j* in year *i*; and *DN*_*ij*_ represents the aggregate brightness value for province *j* in year *i*; *α*_*ij*_ represents the per capita carbon emissions for province *j* in year *i*, and *β*_*ij*_ denotes the carbon emissions per unit of DN value for province *j* in year *i*. Utilizing ArcGIS 10.6 software to perform the natural breaks method for classifying *α*_*ij*_ and *β*_*ij*_ respectively, The integrated *α*_*ij*_ and *β*_*ij*_ can be categorized into three groups, encompassing a total of 59 datasets for 14 cities over a span of four years. and the grouping is presented in Table 3.

**Table 3:** Data grouping situation.

Grouping type	Year	Subgroup affiliation data
AA type	2005	Ili、Bortala、Kizilsu、Kashi、Hotan
	2010	Ili、Bortala、Kizilsu、Kashi、Hotan
	2015	Turpan、Bortala、Bayingolin、Aksu、Kizilsu、Hotan、Kashi、Ili、Tacheng、Altay
	2020	Turpan、Bortala、Kizilsu、Bayingolin、Aksu、Hotan、Kashi、Ili、Tacheng、Altay
AB type	2005	Changji、Hami、Turpan、Tacheng、Bayingolin、Aksu、Altay
	2010	Changji、Hami、Turpan、Tacheng、Bayingolin、Aksu、Altay
	2015	Karamay、Changji、Hami
	2020	Karamay、Changji、Hami
BA type	2005	Urumqi、Karamay
	2010	Urumqi、Shihezi
	2015	Urumqi、Karamay、Shihezi
	2020	Urumqi、Karamay、Shihezi

**Note:** Calculations include data on the use of night-time light, energy consumption statistics and population data; information on Shihezi for 2005 is not available.

This research employs three unique datasets of carbon emissions spanning from 2005 to 2020. The analysis starts by inputting the consolidated carbon emission figures into SPSS to perform a linear regression analysis, excluding an intercept term [25]. The regression equation parameters for carbon emissions are presented in Table 4, the computation is as follows:

$$z=ax_{1}+bx_{2}$$ (11)

**Table 4:** The parameters of the quadratic polynomial model for carbon emissions.

Model	*a*	*b*	R^2^
11	-0.262	0.750	0.965
12	-0.111	0.385	0.951
21	0.039	-0.375	0.916

The formula used for estimating carbon emissions is as follows. *z* representing the estimated carbon emissions, is calculated using regression coefficients *a* and *b* for each independent variable, with *x*_*1*_ denoting the total nighttime light metric value, and *x*_*2*_ signifying the population data.

### 3.3 Analysis of spatial autocorrelation

#### 3.3.1 Global autocorrelation

Global spatial autocorrelation evaluates the relationship between the spatial pattern of a given attribute and its importance within the whole are, as well as the homogeneity of attribute values in proximate regions. The range of values for Moran’s I index falls within [–1,1] [26], the computation is as follows:

$$Global\;Moran's\;I=\frac{n\sum_{i=1}^{n}\sum_{j=1}^{n}w_{ij}(x_{i}-\tilde{x})(x_{j}-\tilde{x})}{\sum_{i=1}^{n}\sum_{j=1}^{n}x_{ij}{\left(x_{i}-\tilde{x}\right)}^{2}}$$ (12)

where *n* signifies the number of city samples, represents the carbon emission status of a city, and *x*_*i*_ and *x*_*j*_ represents the carbon emission status of cities *i* and *j*, respectively. *w*_*ij*_ represents the spatial weight matrix for county *i* and *j*.

#### 3.3.2 Local autocorrelation

Local autocorrelation is utilized to appraise the distributional traits of individual spatial entities within the analysis region and their interactions with adjacent entities, culminating in the determination of the extent of spatial association [25], the computation is as follows:

$$Local\;Moran's\;I=\frac{n(x_{i}-\tilde{x}(\sum_{j=1}^{n}w_{ij}(x_{j}-\tilde{x})}{\sum_{i=1}^{n}{\left(x_{i}-\tilde{x}\right)}^{2}}$$ (13)

With *n* representing the number of neighboring municipalities to municipality *I*, and the other variables defined as before. A positive value of the *Local Moran’s I* index signifies a high-high (HH) clustering spatial pattern between the city and its neighbors. The *Moran’s I* index quantifies the spatial autocorrelation of carbon emissions among cities. A negative *Moran’s I* index indicates dissimilarities in the spatial clusters between a city and its neighboring cities, exhibiting a low-low (LL) clustering pattern.

### 3.4 Change frequency analysis

In this paper, the 4-year energy carbon emission data of Xinjiang region from 2005 to 2020 are statistically analyzed by using digital coding method, followed by the difference calculation of the 4 periods of energy carbon emission estimation results of Xinjiang region from 2005 to 2020 using grid calculator, with the obtained carbon emission estimation results calculated by using mathematical coding operation method. Finally, the grid map of the change frequency of Xinjiang energy carbon emissions from 2005 to 2020 is obtained [27]. the computation is as follows:

$$code_{05-20}=100\times code_{05-10}+10\times code_{10-2015}+1\times code_{15-2020}$$ (14)

In the formula, *code*_*05-20*_ indicates the frequency code of energy carbon emission change in Xinjiang region from 2005 to 2020, and *code*_*05-10*_, *code*_*10-15*_ and *code*_*15-20*_ indicate the code of 3-period difference (0 or 1), respectively, with a total of 2 categories and 14 types of change frequency codes, as presented in Table 5.

**Table 5:** Statistics of carbon emissions change frequency.

Frequency code	Classification value	Type
0000	0	Unchanged
0001、0010 and 0100	1	One change occurs
0011、0101、1001、0110 and 1010	2	Secondary changes occur
1111	3	Three changes occurred

### 3.5 Standard deviation ellipse analysis

In 1926, the famous scholar Lefever proposed a method of calculating data using spatial statistics called Standard Deviation Ellipse (SDE), which reflected the variation trend by using ellipse changes and gravity center forward [28]. This quantitative approach to spatial statistics is extensively employed in the examination of space-time distribution patterns [29]. The formulae for each parameter are presented as follows:

$$\left(\bar{X},\bar{Y})=(\frac{\sum_{i=1}^{n}w_{i}x_{i}}{\sum_{i=1}^{n}w_{i}},\frac{\sum_{i=1}^{n}w_{i}y_{i}}{\sum_{i=1}^{n}w_{i}}\right)$$ (15)

$$\tan\theta =((\sum_{i=1}^{n}w_{i}^{2}{\tilde{x}}_{i}-\sum_{i=1}^{n}w_{i}^{2}{\tilde{y}}_{i}^{2}+\sqrt{{\left(\sum_{i=1}^{n}w_{i}^{2}{\tilde{x}}_{i}^{2}-\sum_{i=1}^{n}w_{i}^{2}{\tilde{y}}_{i}^{2}\right)}^{2}-4\sum_{i=1}^{n}w_{i}^{2}{\tilde{x}}_{i}^{2}{\tilde{y}}_{i}^{2}})/2\sum_{i=1}^{n}w_{i}^{2}{\tilde{x}}_{i}{\tilde{y}}_{i})$$ (16)

$$\sigma_{X}=\sqrt{\frac{\sum_{i=1}^{n}{\left(w_{i}{\tilde{x}}_{i}\cos\theta -w_{i}\tilde{y}\sin\theta \right)}^{2}}{\sum_{i=1}^{n}w_{i}^{2}}}$$ (17)

$$\sigma_{y}=\sqrt{\frac{\sum_{i=1}^{n}{\left(w_{i}{\tilde{x}}_{i}\sin\theta -w_{i}{\tilde{y}}_{i}\cos\theta \right)}^{2}}{\sum_{i=1}^{n}w_{i}^{2}}}$$ (18)

In the equations above, $\bar{X}$ and $\bar{Y}$ represents the weighted mean center of the ellipse; *x* and *y* represent the gravity coordinates of the spatial location of the ellipse; and the rotation angle *θ* represents the included Angle formed by the deviation of the center coordinate of the elliptic element. The *w*_*i*_ represents the weight of the cell of the ellipse; *σ*_*x*_, *σ*_*y*_ represents the standard deviation of the element of the ellipse along the *x* and *y* axes.

### 3.6 Technical route

**Fig 3:**
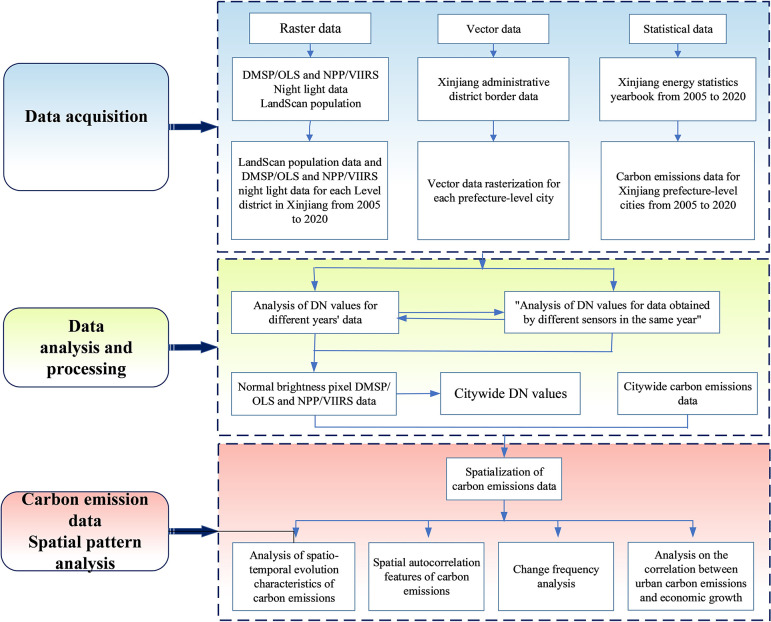
Technical route.

The technical route includes main components and steps of the study. The first part includes data collection: DMSP/OLS and NPP/VIIRS data, statistical data, and administrative vector data. In the data analysis and processing phase, a long time-series fusion data was constructed using NPP and DMSP, and a simulation model was built between the corrected night-light data from 2005 to 2020 and the known carbon emission data in the study area. In the analysis stage, we explored the spatial variation characteristics of carbon emissions in Xinjiang and analyzed the city-scale spatial distribution characteristics as well as the relationship between it and economic development. Finally, specific measures for carbon reduction were proposed based on the above ana,. The specific technical route is shown in Fig 3.

## 4 Result and analysis

### 4.1 Evaluation of the carbon emission estimation results

**Fig 4:**
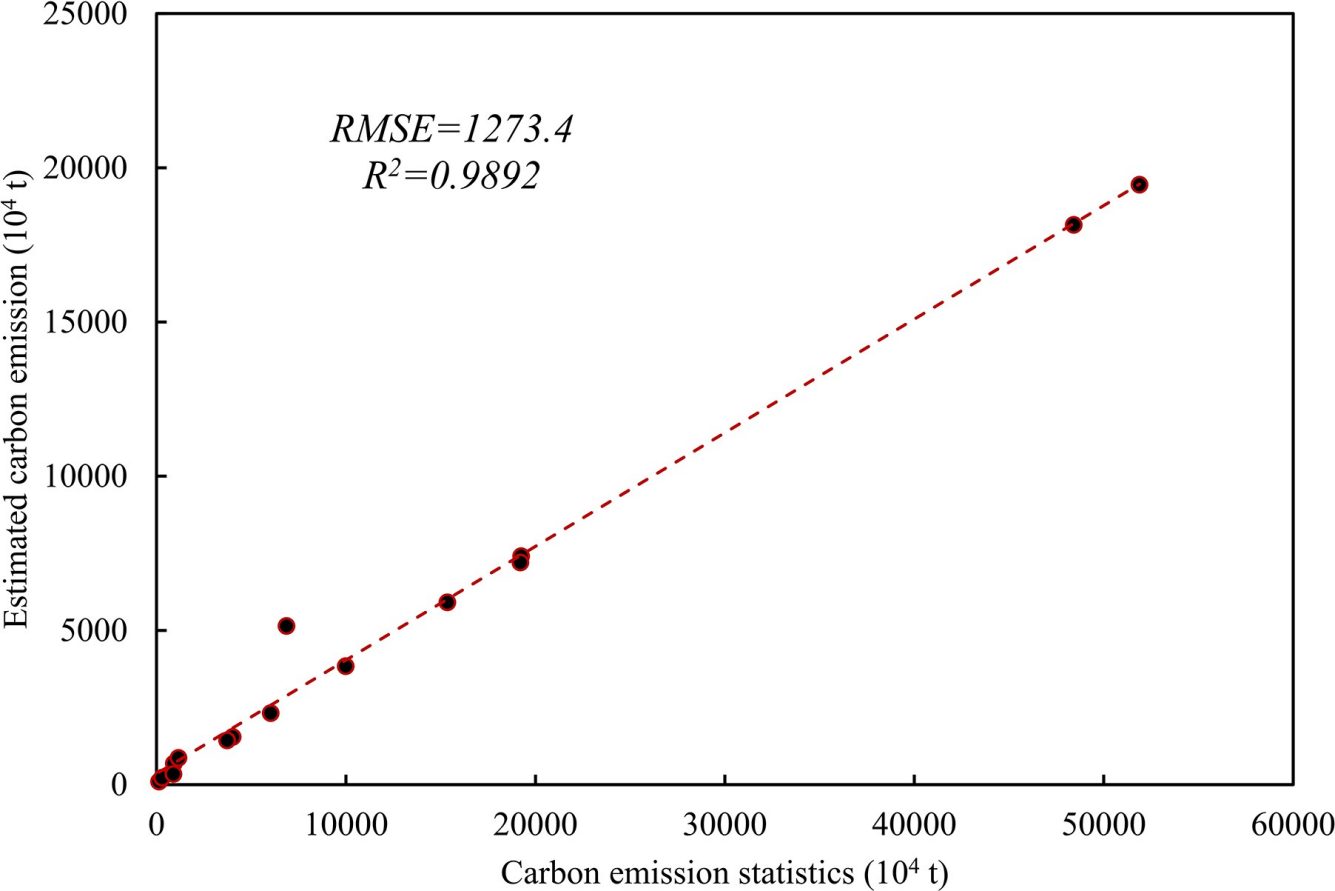
Verification of carbon emission simulation accuracy based on statistics.

According to statistical analysis, Xinjiang’s energy carbon emissions displayed distinct phased fluctuations, which were primarily reflected in the growth rate from 2005 to 2010 and 2010 to 2015, amounting to 54.2% and 22.7%, respectively. Notably, the growth rate from 2015 to 2020 was 47.6%. Simultaneously, it shows that the period of fastest growth is from 2005 to 2010. Consequently, this study selects the year 2005 as the initial point to validate the precision of the model and employs regression analysis on the carbon emission data of the 14 prefecture-level cities in Urumqi (Fig 4). The results show that the R^2^ of 2005 is 0.9983, and the RMSE is 1491.504. This indicates a robust positive correlation between night light data and the carbon emission estimation model, validating its applicability for analyzing the temporal and spatial variations of energy consumption-related carbon emissions in Xinjiang.

### 4.2 Examination of the temporal and spatial fluctuations of carbon emissions

#### 4.2.1 Spatial and temporal distribution of carbon emissions

Table 6 demonstrates that the total energy carbon emission in Xinjiang has been consistently increasing from 2005 to 2020, Nevertheless, the growth rate of carbon emission displays a downward trend. From 6.02×10^8^ tons in the year 2005 to 11.99×10^8^ tons in the year 2020, the total carbon emission increased. Nevertheless, the average annual growth rate decreased from 80.3% in 2005-2010 to 40.4% in 2015-2020. These figures directly reflect China’s economic development requirements for energy transformation, resulting in a deceleration of the growth rate of carbon emissions from energy consumption in Xinjiang.

**Table 6:** Carbon emission from 2005 to 2020 in Xinjiang.

Projects	2005	2010	2015	2020
Carbon emission/10^8^ t	6.02	10.86	8.53	11.99
Average annual growth rate/%	-	80.3	27.3	40.4

**Fig 5:**
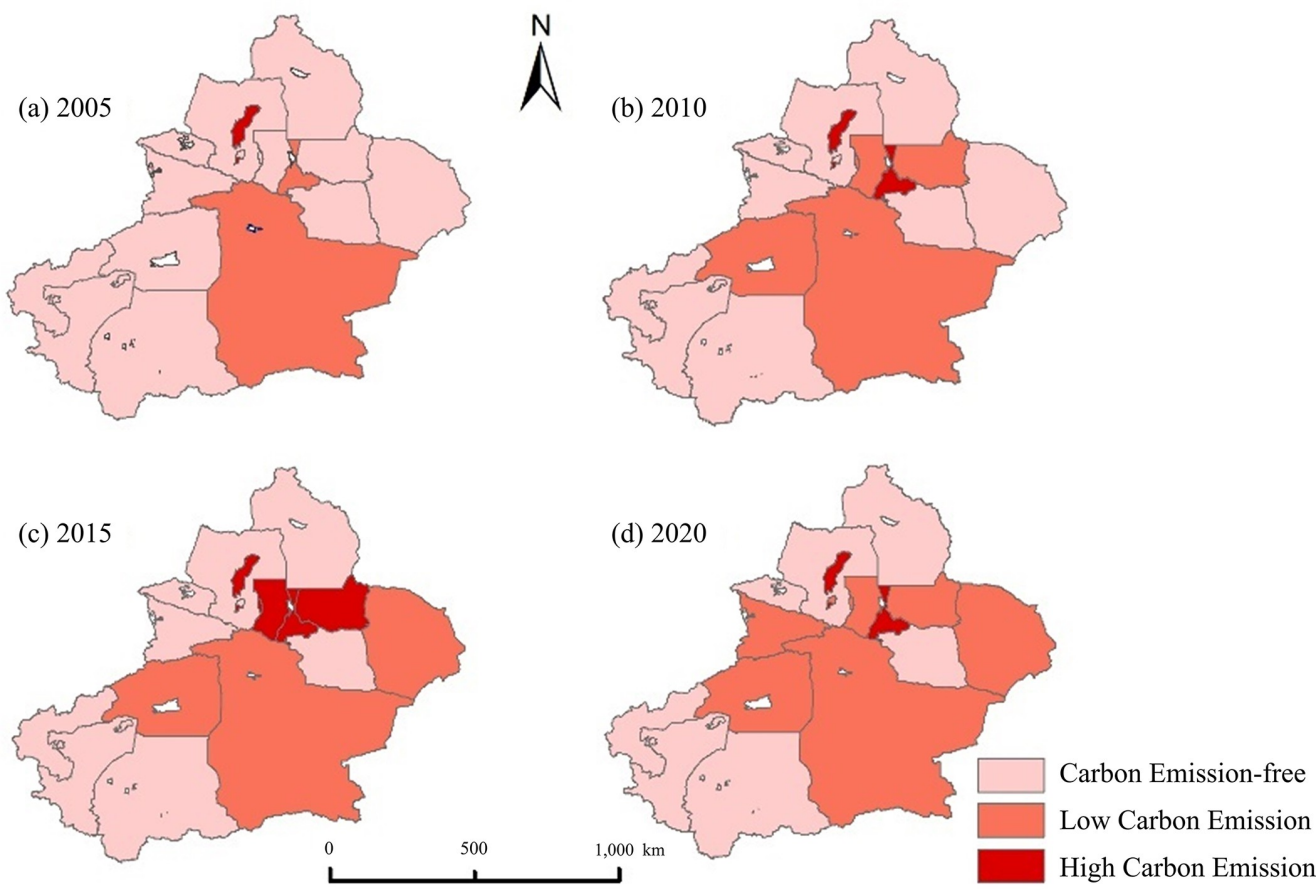
The spatial distribution of carbon emissions in Xinjiang from 2005 to 2020 (GS (2024)0650).

This investigation applied ArcGIS for data analysis and showcased the spatial arrangement of carbon emissions in Xinjiang (Fig 5). The primary approach was to classify the estimated results into three groups, including those with no carbon emissions (< 0×10^6^ t), low carbon emissions (0~1.16×10^6^ t), and high carbon emissions (> 1.16×10^6^ t). The findings reveal that areas with no carbon emissions are primarily situated in the northwest and southwest sections of Xinjiang, with regions of low carbon emissions subsequent in the expansive small and medium-sized urban zones. On the contrary, areas characterized by high carbon emissions are mainly situated near provincial capitals and urban clusters. Regions without inhabitants or with low populations emit less carbon, whereas the primary carbon emission areas are centered on key cities and their surrounding densely populated industrialized regions [30]. In Xinjiang, carbon-free areas are mainly distributed in Altay, Tacheng, and Bozhou in the northwest, and Hotan, Kashgar, and Kezhou in the southwest, low-carbon emission areas are clustered in sizable small and medium-sized urban regions, including Aksu Prefecture, Bayingoleng Mongolian Autonomous Prefecture, Hami City, and Ili Prefecture. In contrast, zones with high carbon emissions are concentrated around provincial capitals, particularly in Urumqi, Changji, Shihezi and Karamay.

#### 4.2.2 Spatial autocorrelation of carbon emissions

**Fig 6:**
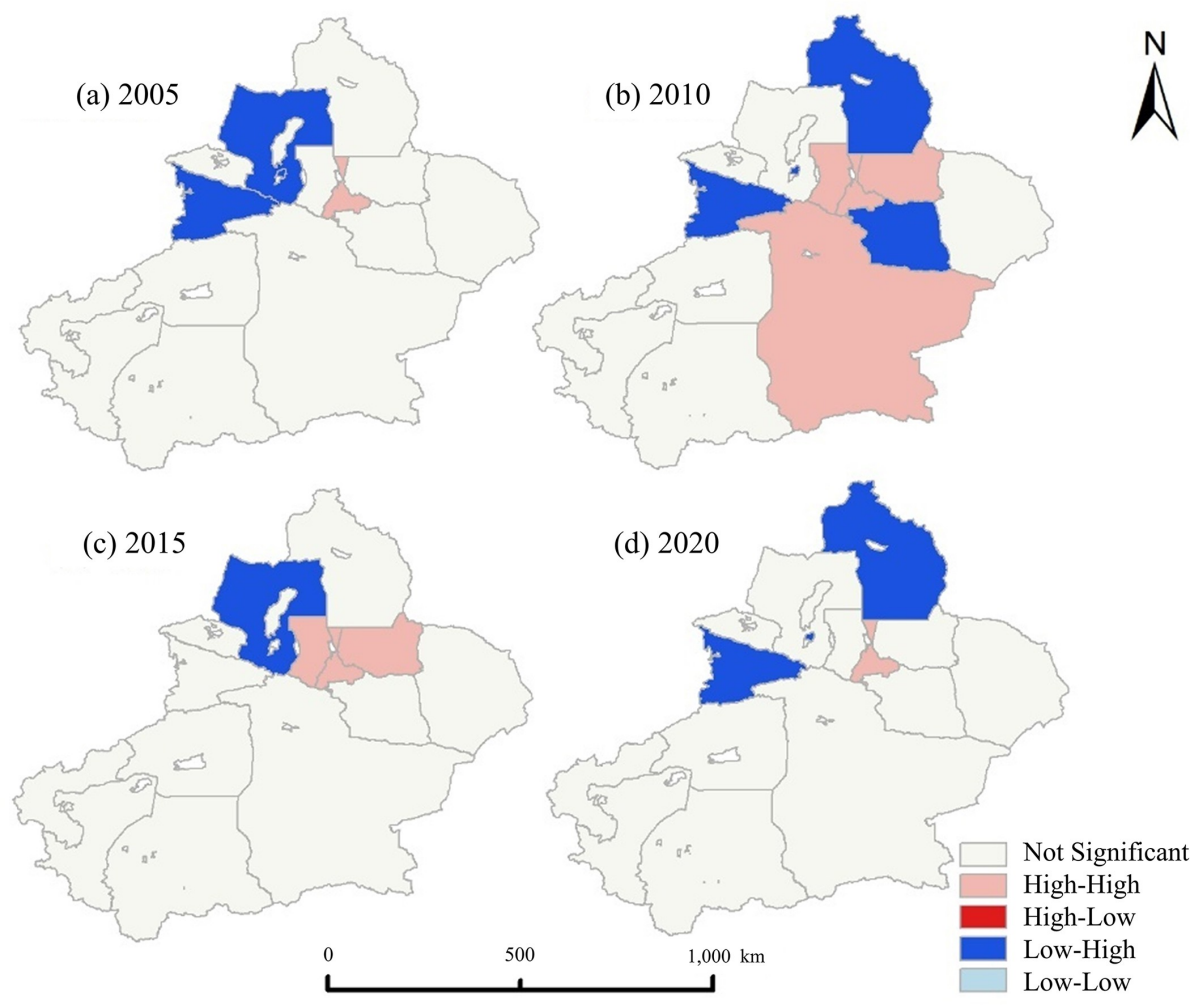
Cluster conditions of the carbon emissions from 2005 to 2020 in Xinjiang (GS (2024)0650). **Note:** It includes data on Kashgar’s GDP and Shihezi’s energy-related carbon emissions for the year 2005, which are presently unavailable.

The global Moran’s I index was used to assess the spatial autocorrelation of energy carbon emissions in Xinjiang (Fig 6). Upon analyzing carbon emissions in Xinjiang, it was observed that all index values surpassed 0, suggesting a propensity for either high or low value clustering of energy-related carbon emissions within the region, trending towards stability [31]. The results indicate that the local spatial autocorrelation of energy carbon emissions in Xinjiang is characterized by high-high concentration (HH) and low-high concentration (LH). Between 2005 and 2020, the number of cities with low-high concentration of carbon emissions in Xinjiang showed a marked increase, with an expanding scale. Meanwhile, the number of cities with high-high concentration in the Tacheng region and Yili Prefecture gradually decreased. In 2005, a high-high agglomeration area was formed in Urumqi region, and a low-high agglomeration area was formed in Yili Kazak Autonomous Prefecture and Tacheng region. By 2010, the scale of low-high aggregation and high-high aggregation in Xinjiang cities had increased, resulting in the formation of high-high agglomeration areas in Urumqi, Changji, and Bayingoleng Mongolian Autonomous Prefecture, while low-high aggregation areas were formed in Turpan, Altay, and Yili Prefecture. However, by 2015, the scale of high-high agglomeration areas and low-low agglomeration areas in Xinjiang had further decreased, with Urumqi and Changji formed a high-high agglomeration area, while only one low-high agglomeration area was formed in Tacheng region. By 2020, there was a new low-high concentration area in Altai and Yili prefecture, and the high-high concentration area in Urumqi, while the carbon emission space of other regions did not change significantly.

### 4.3 Carbon emission change frequency analysis

**Fig 7:**
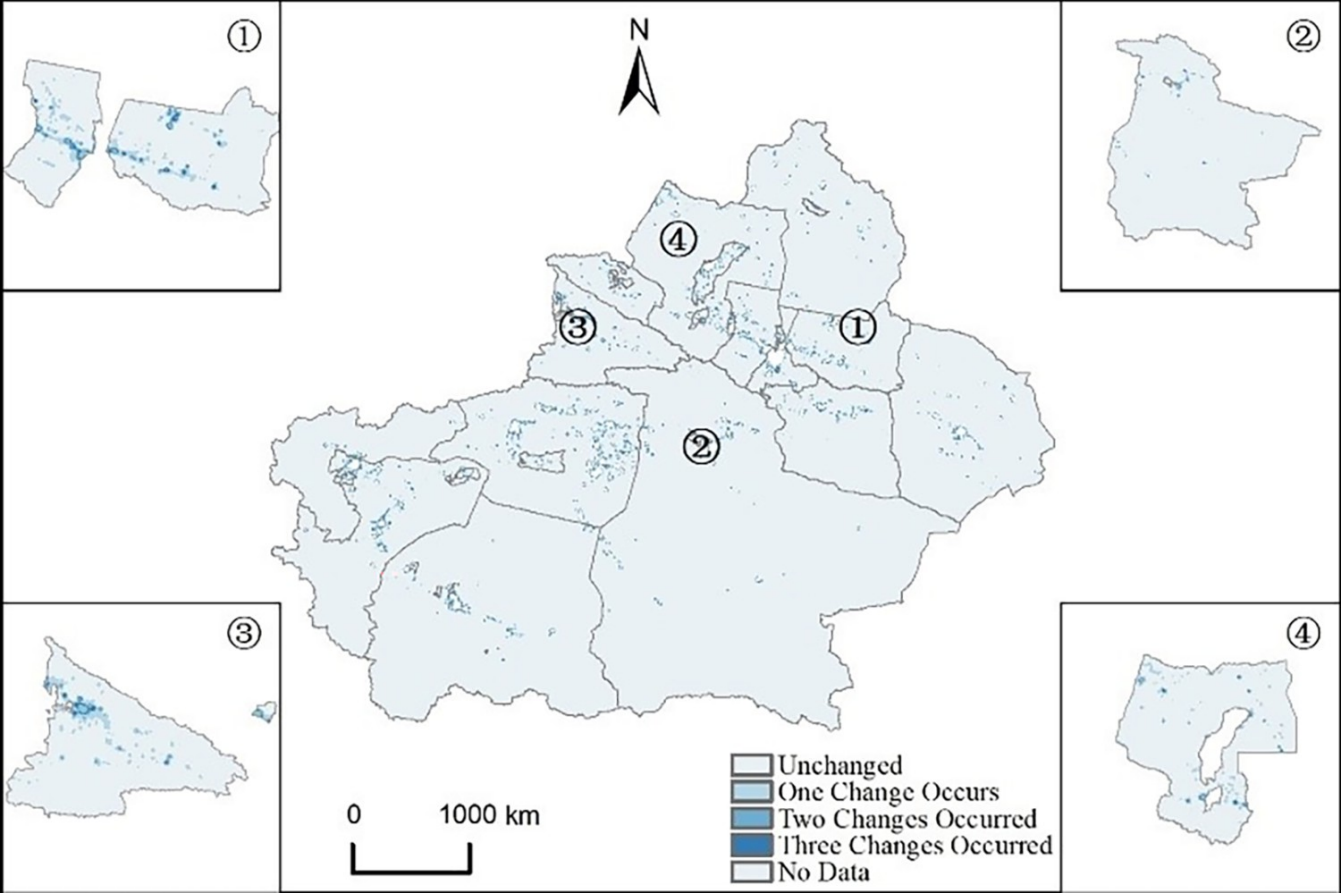
The figure depicts the changes in energy-related carbon emissions in Xinjiang from 2005 to 2020 (GS (2024)0650), where the boundaries represent the division between central urban areas and municipalities, indicating (① Changji Hui Autonomous Prefecture; ②Bayingolin Mongol Autonomous Prefecture; ③ Ili Kazakh Autonomous Prefecture; ④ Tacheng).

This paper uses digital coding method to conduct a quantitative analysis of the change in Xinjiang’s energy carbon emissions (Fig 7). The findings indicate that the carbon emission levels in Xinjiang’s non-carbon and low-carbon emission areas have not experienced substantial shifts, maintaining a relatively consistent level of carbon emissions. These areas are mainly provincial capitals and key cities, with largely stable urban spatial and industrial structures. Concurrently, due to the high-carbon emission enterprises being primarily situated on the city’s outskirts, there are no enterprises located in the central urban area. As a result, carbon emissions in the central urban area remain relatively stable. This highlights that regions where carbon emissions change are primarily determined by the location of high-carbon emissions, which are predominantly distributed in urban areas and suburban locations outside provincial capitals and key cities. The observations suggest that the alteration frequency of urban carbon emissions is predominantly influenced by the geographical distribution of emission sources.

### 4.4 Analysis of elliptic center of gravity migration of carbon emission

**Fig 8:**
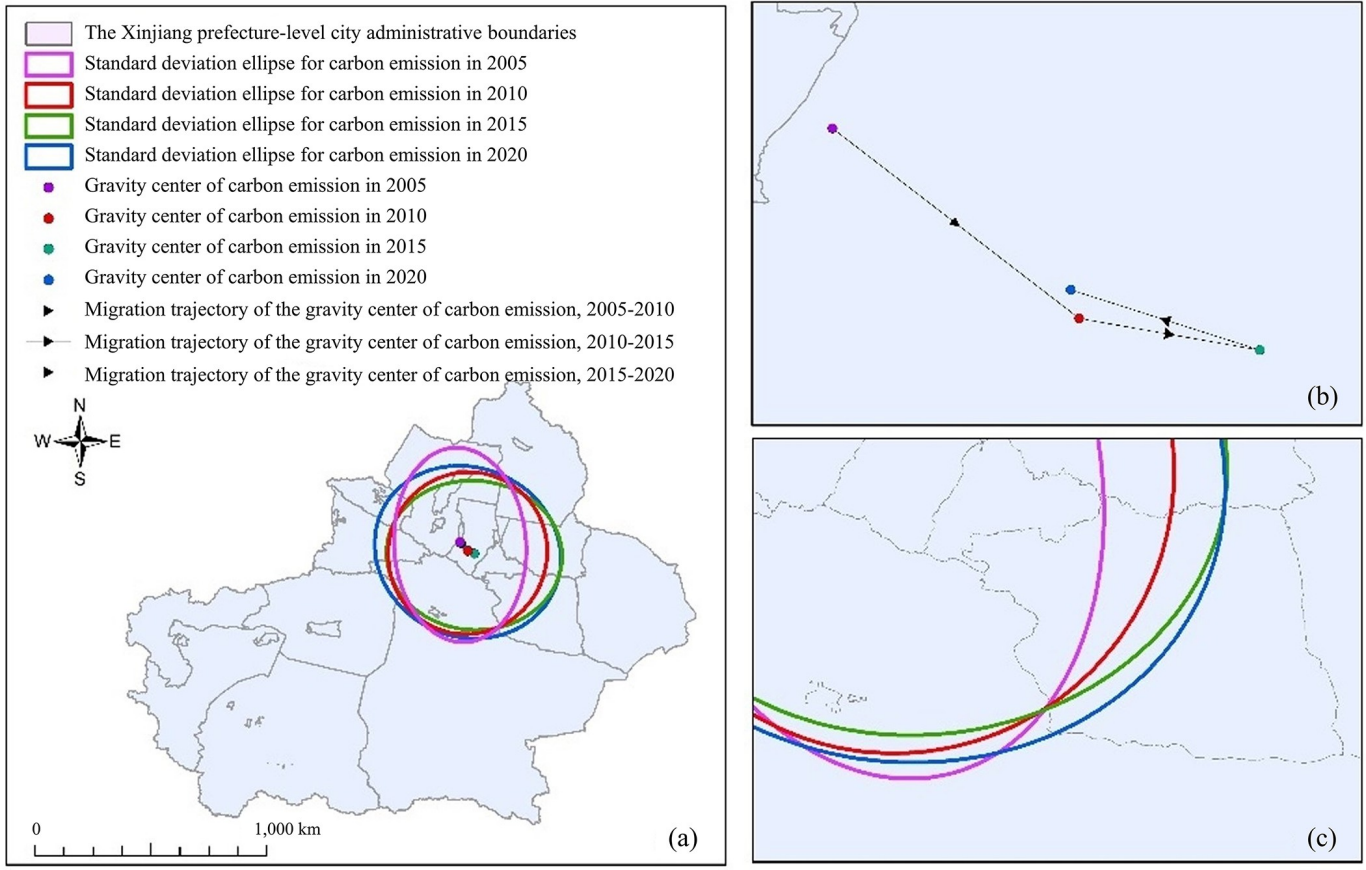
Carbon emissions standards deviation ellipse of provinces 2005 to 2020 in Xinjiang (GS (2024)0650), (a) Carbon emissions standards deviation ellipse distribution diagram, (b) Carbon emissions center of gravity migration locus, (c) Carbon emissions standards deviation ellipse.

This paper adopts the SDE method for complementary analysis [32,33]. It can be observed from Table 7 and Fig 8 that the carbon emission gravity center of Xinjiang varies between 84°21’22.47"-85°13’28.95" E, 42°11’26.49-342°37’45.76" N in the last 15 years. The range of the standard deviation ellipse for Xinjiang’s energy carbon emissions from 2005 to 2020 exhibited a general expansion, yet it contracted in 2015, which is consistent with nationwide policies promoting energy conservation and emission reduction.

**Table 7:** The evolution of the standard deviation ellipse model parameters for Xinjiang’s provinces.

Year	Center coordinates		Transfer of the gravity center		Major axis standard deviation (km)	Minor axis standard deviation (km)	Rotation angle (°)
	Longitudes (°)	Longitude (°)	Orientation	Distance (km)			
2005	86°04′47.34″	43°57′53.85″	-	-	313.48	454.57	164.63
2010	86°28′25.01″	43°45′25.77″	Southeast	40.53	374.41	378.91	36.43
2015	86°46′00.38″	43°43′32.18″	Southeast	23.80	416.16	346.45	84.53
2020	86°27′44.49″	43°47′12.89″	Northwestern	25.79	449.24	392.61	101.64

The rotation angle θ fluctuates between 164.63° and 101.64° in the period from 2005 to 2020, this suggests a shift in the distribution of energy-related carbon emissions within Xinjiang, moving from the southeastern regions towards the northwestern areas during the preceding 15 years. From 2005 to 2010, the rotation Angle decreased from 164.63° to 36.43°. As the rotation Angle decreased, the movement of carbon emissions from southeast to northwest in Xinjiang slowed down over the past 15 years. From 2010 to 2015, the rotation angle expanded from 36.43° to 84.53°, and the spatial pattern shifted from southeast to northwest with an increase in the rotation angle. From 2015 to 2020, The spatial pattern of energy carbon emissions shifted from northwest to southeast, with the rotation angle θ increasing from 84.53° to 101.64° over the course of 15 years. The shift of center of gravity gradually strengthened the spatial pattern from northwest to southeast, forming a trend.

The discernible pattern in the spatial distribution of energy carbon emissions along the principal and minor axes from 2005 to 2020 reveals a distinct directional trend. This points to a distribution pattern of energy consumption-related carbon emissions in Xinjiang, which are dispersed from the northeast towards the southwest. Indicating a strong diffusion trend overall, A clear polarization phenomenon is observed in the elliptical standard deviation of carbon emission distribution from northwest to southeast in 2010, decreased from 374.41 km to 416.16 km in 2015.Over the course of 15 years, The consistent decline in energy-related carbon emissions observed from the northwest to the southeast in Xinjiang is demonstrated by the expansion of the elliptical standard deviation along the axis of carbon emission distribution. Furthermore, there was an increase in the range of carbon emissions from energy consumption and a narrowing of the standard deviation of the short axis. The directional movement of carbon emissions stemming from energy use also exhibited a pronounced westerly trend, indicating a potential transition to more sustainable energy sources.

### 4.5 The correlation between urban carbon emissions and economic growth

**Fig 9:**
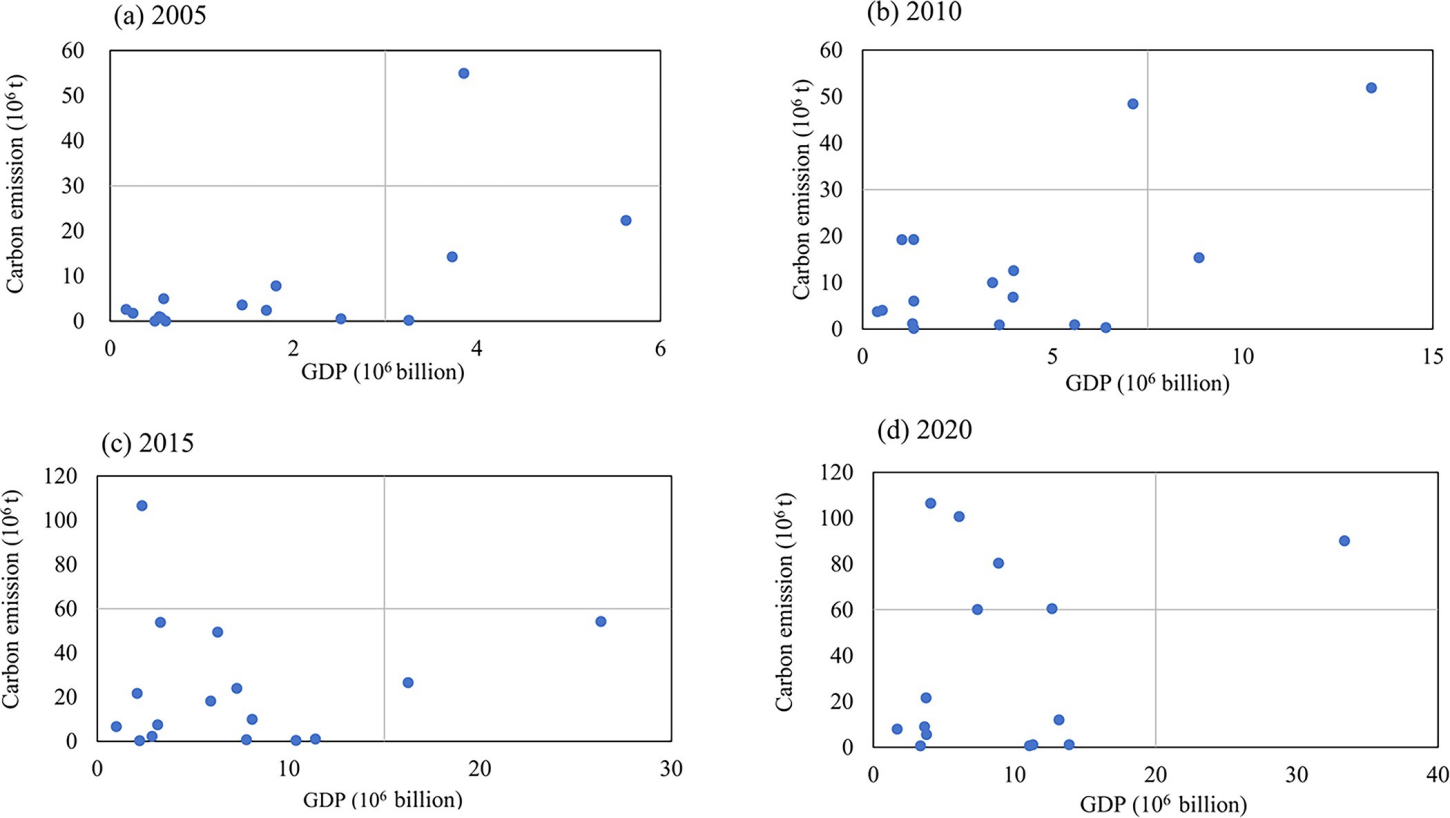
The correlation between carbon emissions and economic growth in Xinjiang from 2005 to 2020.

The verification method first assesses the correlation between urban carbon emissions and economic growth in Xinjiang for the period from 2005 to 2020. It then divides the gross urban product (GDP) and urban carbon emissions into two distinct dimensions, this research used a quartile map to investigate the relationship between urban development, energy consumption, and economic growth (Fig 9). The graph shows a relationship between the carbon emissions of 14 cities and the gross regional product. The horizontal axis represents the gross regional product, while the vertical axis represents the carbon emissions of the region. The first to fourth quadrants represent "high energy-high income" cities, "high energy-low income" cities, "low energy-low income" cities, and "low energy-high income" cities, respectively. The distinct economic conditions directly influence and restrict energy consumption and carbon emissions, while energy consumption and carbon emissions also have an impact on urban economic development [34].

## 4 Discussion and conclusion

### 4.1 Discussion

In this paper, we applied the DMSP-OLS and NPP-VIIRS nighttime light data to estimate the energy-related carbon emission in Xinjiang from 2005 to 2020. The results demonstrated that integrated nighttime light data can efficiently estimate energy-related carbon emissions [35–37]. Accordingly, we analyzed in detail the spatial and temporal evolution characteristics of energy carbon emissions in Xinjiang and evaluated the relationship between carbon emissions and economic development, thus providing a scientific theoretical basis for low-carbon development in Xinjiang. Furthermore, the growth rate of carbon emissions showed a decreasing trend throughout the study period, which is supported by the findings of Zhang et al. [38]. From 2005 to 2020 in Xinjiang, energy-related carbon emissions showed significant variations and were closely linked to economic development, which is consistent with the findings of Qin et al. [39]. The restructuring of industrial structures and the shift towards a low-carbon economy in Xinjiang are conducive to the growth of low-carbon energy structures [40]. However, it must be pointed out that compared with national and prefecture-level carbon emission studies, inversion of carbon emission using nighttime light data may lead to deviations from linear simulation, with relatively uncertain results. Thus, this paper still has shortcomings, including in the following aspects. Firstly, as the selected energy types did not cover all types and there was no data with city, the research’s outcomes influenced the characteristics of spatial and temporal distribution patterns to some extent. The estimated carbon emissions resulting from energy consumption exhibited a discrepancy compared to the actual carbon emission figures. Secondly, the carbon emission from energy consumption is a continuous and long-term process, leading to data biases caused by external environmental factors [41]. Therefore, with the enhancement and improvement of the availability of basic data, future research methods can further improve relevant research from the aspects of sensitivity analysis, uncertainty analysis model construction, index selection, spatial measurement and so on, towards enriching results [42]. Thirdly, as the share of new energy consumption progressively rises, the carbon emission data for wind energy, solar energy, nuclear energy, and other new energy sources have shifted. This has led to skewed carbon emission estimation outcomes, which are contingent upon the aggregate brightness values derived from night-time lighting data [43]. In this respect, addressing the issue of nighttime light data total brightness value deviation by finding solutions should be prioritized in future research.

### 4.2 Conclusion

This paper utilized the fused DMSP-OLS and NPP-VIIRS data to construct a long time-series nighttime lighting dataset and estimated energy carbon emissions in Xinjiang using the IPCC-recommended method. Based on this, we assigned the carbon emission data to the image scale so as to analyze its spatial distribution characteristics using spatial autocorrelation and standard deviation ellipse, and finally analyzed the relationship between energy carbon emission and economic development based on the quartile diagram. The results of this study have led to several conclusions:

1. From 2005 to 2020, carbon emissions in Xinjiang increased continuously, with high emissions concentrated in provincial capitals and lower emissions scattered in smaller cities. However, growth has slowed due to policies aimed at reducing emissions.
2. The urban carbon emissions in Xinjiang continue to rise, with obvious regional differences in 2005-2020. The urban “low-high” and “high-high” energy consumption patterns are prominent, with the overall characteristics remaining stable.
3. Carbon emissions in Xinjiang’s non-emission and low-emission areas have remained stable, with central cities as the main carbon emission centers, forming a circular pattern within the Economic Belt and surrounding areas.
4. There are some changes in the spatial distribution of carbon emissions in Xinjiang, with its standard deviation ellipse and the center of gravity all shifted to the southeast. Such performance is closely related to economic development.

## Supporting information

S1 File (ZIP)
